# Synergistic Inhibition of Porcine Reproductive and Respiratory Syndrome Virus by a Bifunctional 5′-PPP miRNA Combining RIG-I Activation with Sequence-Specific Viral Targeting

**DOI:** 10.3390/v18030390

**Published:** 2026-03-20

**Authors:** Zihang Song, Jiabao Hou, Feng Guo, Longping Chen, Chudong Wang, Xinjie Guo, Ping Li, Wenlong Shen, Jiajun Yang, Hongxu Zhong, Hanlu Zhang, Yan Zhang, Enqi Du, Zhihu Zhao

**Affiliations:** 1 College of Veterinary Medicine, Northwest A&F University, Xianyang 712100, China; 2 National Key Laboratory of Advanced Biotechnology, Academy of Military Medical Sciences, Beijing 100071, China; 3 Department of Research and Development, Yangling Carey Biotechnology Co., Ltd., Xianyang 712100, China

**Keywords:** 5′-PPP, miRNA, PRRSV, RIG-I

## Abstract

The immunosuppressive nature of porcine reproductive and respiratory syndrome virus (PRRSV) remains the central obstacle to its effective control. Conventional microRNA (miRNA)-based antiviral approaches are limited by their modest potency and the high risk of viral escape. Here, we rationally designed an engineered miRNA carrying a 5′-triphosphate (5′-PPP) terminus that integrates RIG-I-driven innate immune activation and sequence-specific gene silencing within a single molecule. In vitro-transcribed 5′-PPP miRNAs are efficiently recognized by the pattern-recognition receptor RIG-I, triggering a robust type I interferon response that counteracts PRRSV-induced immunosuppression. In MARC-145 cells, one such construct, 5′-PPP BZL-sRNA-20, potently inhibited PRRSV replication through the synergistic action of immune activation and gene silencing. However, in porcine alveolar macrophages (PAMs)—the natural host cells for PRRSV—the antiviral effect depended primarily on 5′-PPP-induced interferon responses, with the targeting sequence providing limited or context-dependent benefits. Dual-luciferase assays confirmed that the gene-silencing activity depends on 5′-PPP modification, which enhances the stability of BZL-sRNA-20. This bifunctional strategy establishes an “immune activation plus targeting” paradigm by simultaneously acting as a RIG-I ligand that triggers broad antiviral responses and specifically cleaves viral RNA via direct base-pairing to conserved regions of the PRRSV genome. These findings reveal the potential of engineered 5′-PPP miRNAs as immunomodulatory antiviral agents, while highlighting that the contribution of RNAi targeting varies depending on the cellular context.

## 1. Introduction

Porcine reproductive and respiratory syndrome (PRRS) represent one of the most economically devastating diseases affecting global swine production, with annual losses exceeding USD $1.2 billion in the United States alone [1]. The causative agent, PRRSV, is an enveloped, single-stranded positive-sense RNA virus belonging to the family Arteriviridae [2]. Its approximately 15 kb genome encompasses 11 open reading frames flanked by 5′- and 3′-untranslated regions (UTRs) [3]. The high mutation rate of the viral genome, frequent recombination among different lineages/sub-lineages, and virus mediated immunosuppression, effective preventive measures remain unavailable.

Plant-derived small RNAs (sRNAs) represent a promising class of antiviral agents that directly mediate mRNA cleavage through complementary base-pairing mechanisms [4,5]. Numerous naturally occurring plant sRNAs have demonstrated potent antiviral activity. For example, *honeysuckle*-encoded miR2911 directly targets genomic sequences of influenza A virus, enterovirus 71, and SARS-CoV-2, effectively suppressing viral replication [6,7,8]. A recent study showed that miR2911 also specifically recognizes the PRRSV genome and markedly inhibits viral replication in vitro [9]. In addition, BZL-sRNA-20, isolated from *Scutellaria barbata* (Ban Zhi Lian), has been reported to attenuate pathogen-induced cytokine storms and alleviates acute lung injury by targeting host Toll-like receptor 4 [10]. Nevertheless, single miRNA approaches remain vulnerable to viral escape, as the inherently high mutation rates of RNA viruses enable rapid evolution and allow viral escape from miRNA-mediated silencing [11,12].

The innate immune system constitutes the first line of defense against invading pathogens. Host cells rapidly mount an antiviral state upon the detection of pathogen-associated molecular patterns (PAMPs) by pattern recognition receptors (PRRs) [13]. Retinoic-acid-inducible gene I (RIG-I) is a cytosolic RNA sensor that specifically recognizes 5′-triphosphate (5′-PPP) RNA and triggers downstream interferon responses [14,15]. Exploiting this mechanism, numerous studies have employed 5′-PPP-modified RNAs to reactivate virus suppressed innate immunity and confer broad protection against infection [16,17,18]. However, such molecules deliver only generalized immune stimulation and lack sequence specific targeting of individual pathogens, making precision antiviral effects unattainable.

Here, we present the first integration of 5′-PPP-mediated global immune activation with the miRNA-directed gene silencing within a single artificial molecule. Our leading compound 5′-PPP BZL-sRNA-20 simultaneously triggers RIG-I signaling and markedly reduces *ORF7* expression and viral titers in both MARC-145 and PAM cells. Dual-luciferase reporter assays confirm that, via its 5′-PPP moiety, the molecule triggers innate immunity while simultaneously leveraging sequence complementarity to precisely silence the viral genome, thereby achieving a dual-pathway, synergistic anti-PRRSV effect.

## 2. Materials and Methods

### 2.1. Cells and Viruses

MARC-145 cells were maintained in DMEM supplemented with 10% fetal bovine serum (FBS) and 1% penicillin–streptomycin. Porcine alveolar macrophages (PAMs) were obtained by lung lavage from 6-week-old specific pathogen free (SPF) pigs and cultured in RPMI-1640 containing 10% FBS and 1% penicillin–streptomycin. The PRRSV NADC30-like strain used in this study was provided by Prof. Jing-Yu Wang (Northwest A&F University). This strain was originally isolated from clinical samples collected in 2024 from a pig farm in Shaanxi Province, China, where the herd exhibited typical PRRSV infection symptoms including reproductive failure in sows (abortion and stillbirths) and respiratory distress in piglets. The virus was propagated and titrated in MARC-145 cells in our laboratory. Molecular characterization revealed that the *GP5* gene of this strain shares 93.86% nucleotide sequence identity with the NADC30-like reference strain *JN654459*, and phylogenetic analysis further confirmed its classification as the NADC30-like subtype (Figure S1). This strain was selected for the following reasons: (1) NADC30-like strains have become the predominant circulating PRRSV subtype in China since 2013 [19]; (2) the high sequence homology with the reference strain indicates strong representativeness of current epidemic strains; and (3) its isolation from a clinically affected farm ensures relevant pathogenicity and practical significance for antiviral research.

### 2.2. In Vitro Transcription of 5′PPP miRNAs and 5′PPP-Removal

5′-triphosphate (5′-PPP) miRNAs were produced by in vitro transcription using the HiScribe T7 Quick High Yield RNA Synthesis Kit (New England Biolabs, Ipswich, MA, USA, NEB, E2050S) according to the manufacturer’s instructions. DNA templates were generated by annealing complementary single-stranded oligonucleotides containing a T7 promoter followed by the miRNA sequence (synthesized by Beijing Tianyi Huiyuan Co., Ltd., Beijing, China):

miR-NC (S): 5′-TAATACGACTCACTATAGTTCTCCGAACGTGTCACGTTT-3′
miR-NC (AS): 5′-AAACGTGACACGTTCGGAGAACTATAGTGAGTCGTATTA-3′
miR-181c (S): 5′-TAATACGACTCACTATAGAACATTCAACCTGTCGGTGAGT-3′
miR-181c (AS): 5′-ACTCACCGACAGGTTGAATGTTCTATAGTGAGTCGTATTA-3′
BZL-sRNA-20(S):5′-TAATACGACTCACTATAGGTTCAGAGTTCTACAGTCCGACGATC-3′
BZL-sRNA-20 (AS): 5′-GATCGTCGGACTGTAGAACTCTGAACCTATAGTGAGTCGTATTA-3′

Oligonucleotides were mixed in annealing buffer, heated at 90 °C for 5 min, and cooled to 45 °C at −1 °C/min, as described previously [20]. Overnight transcription was performed at 37 °C, followed by DNase I treatment to remove the DNA template. Transcripts were purified with the Monarch RNA Cleanup Kit (NEB, T2050L), quantified using a NanoDrop™ 2000 spectrophotometer (Thermo Fisher Scientific, Waltham, MA, USA), and resolved on 15% TBE-urea gels. Full-length RNA bands were excised and recovered with the small RNA PAGE Recovery Kit (Zymo Research, Irvine, CA, USA, R1070). Unmodified miRNAs were chemically synthesized by Jiangsu Saisofei Co., Ltd., Wuxi, China and the sequences are listed in Table S1.

To remove the 5′-triphosphate, 500 ng of 5′PPP miRNA was incubated with 1 uL calf intestinal alkaline phosphatase (CIP, NEB, M0525V) at 37 °C for 40 min.

### 2.3. MiRNA Transfection and Viral Infection

Unmodified miRNA mimics and in vitro transcribed single-stranded (ss) miRNAs were transfected into MARC-145 or PAM cells at a final concentration of 20 nM using the TransIT-X2 Dynamic Delivery System (Mirus Bio, Madison, WI, USA, MIR 6000) according to the manufacturer’s instructions, unless otherwise stated for dose–response assays. After 24 h, cells were infected with the PRRSV NADC30-like strain at a multiplicity of infection (MOI) of 0.1. At 24 h post infection, cells and/or culture supernatants were harvested for subsequent analyses.

### 2.4. RT-qPCR

Total RNA was extracted from cells or culture supernatants using the Direct-zol™ RNA Miniprep kit (Zymo Research, R2050), following the manufacturer’s instructions. RNA was reverse-transcribed with the TransScript All-in-One First-Strand cDNA Synthesis SuperMix (Transgen Biotech, Beijing, China, AT341), and quantitative PCR was performed with PerfectStart Visual Green qPCR SuperMix (Transgen Biotech, AQ621). *β-actin* served as the endogenous reference, and relative gene expression was calculated by the 2^−ΔΔCt^ method. Primers used for qPCR are listed in the Table 1 below.

For PRRSV quantification in culture supernatants, a plasmid containing the *ORF7* sequence was serially diluted to generate an external standard curve, as previously described [21]. Viral genome copies in each sample were calculated by interpolating the corresponding Ct value against this standard curve.

### 2.5. ELISA

IFN-β levels in MARC-145 cell culture supernatants were determined using the Monkey IFN-β ELISA Kit (Finetest, Wuhan, China EMK0029). Briefly, standards and samples were added to antibody-coated microtiter plates and sequentially incubated with biotinylated detection antibody, HRP-conjugated streptavidin, TMB substrate, and stop solution. Absorbance was measured at 450 nm using a microplate reader, and IFN-β concentrations were calculated from the standard curve.

### 2.6. Western Blot

Cells were harvested and lysed in RIPA buffer freshly supplemented with protease and phosphatase inhibitors. Proteins were resolved on 12% SDS-PAGE gels and transferred onto PVDF membranes. After blocking with Quick Blocking Buffer (NCM Biotech, Suzhou, China, P30500) at room temperature, membranes were incubated overnight at 4 °C with primary antibodies against β-actin (MBL, Nagoya, Japan, M177-3) or PRRSV N (GeneTex, Irvine, CA, USA, GTX129270). Membranes were washed three times with TBST, incubated for 1 h with HRP-conjugated secondary antibodies, washed again, and developed by evenly overlaying ECL substrate (Epizyme Biomedical, Wuhan, China, SQ201). Chemiluminescent signals were acquired with an imaging system.

### 2.7. Immunofluorescence Assay

Cells were fixed with 4% paraformaldehyde for 20 min, washed three times with PBS, and permeabilized with 0.1% Triton X-100 (Yeasen, Shanghai, China, 20107ES76) in PBS for 20 min. After three additional PBS washes, non-specific binding was blocked with 10% goat serum for 1 h. Cells were then incubated overnight at 4 °C with PRRSV-N primary antibody (GeneTex, Irvine, CA, USA, GTX129270), followed by three PBS washes and 1 h incubation at room temperature with FITC-conjugated secondary antibody (Servicebio, Wuhan, China, GB22403) protected from light. Nuclei were counterstained with DAPI (Beyotime, Shanghai, China, C1006), and images were acquired using a digital confocal imaging system (Thermo Fisher Scientific, Waltham, MA, USA, EVOS M7000).

### 2.8. miRNA Target Prediction and Conservation Analysis

Potential BZL-sRNA-20 target sites within the PRRSV genome were predicted with RNAhybrid (https://bibiserv.cebitec.uni-bielefeld.de/rnahybrid/ accessed 20 November 2025)) using a minimum free-energy threshold of −25 kcal mol^−1^. Conservation of the predicted target sequences among representative PRRSV strains was assessed with MEGA 12.0, and a phylogenetic tree was constructed from the aligned sequences.

### 2.9. Dual-Luciferase Reporter Assay

High-conservation BZL-sRNA-20 target sites identified within the PRRSV genome were tandemly concatenated and cloned between the SacI and XbaI sites of the pmirGLO vector (Promega, Madison, WI, USA, E1330) to generate pmirGLO-PRRSV. MARC-145 cells were co-transfected with the indicated miRNA and pmirGLO-PRRSV plasmid; after 48 h, firefly and Renilla luciferase activities were measured with the Duo-Lite Luciferase Assay System (Vazyme, Nanjing, China, DD1205) according to the manufacturer’s instructions.

### 2.10. Cell Viability Assay

Cell viability was assessed using the Cell Counting Kit-8 (Dojindo, CK04) according to the manufacturer’s instructions. Briefly, porcine alveolar macrophages (PAMs) were seeded in 96-well plates at a density of 1 × 10^5^ cells per well. After treatment with the indicated reagents for specified time periods, 10 μL of CCK-8 solution was added to each well and incubated at 37 °C in the dark for 2 h. The absorbance at 450 nm was measured using a microplate reader.

### 2.11. Northern Blot

Northern blotting was performed as previously described [22]. Briefly, 5 µg of total RNA was separated on a 15% TBE-urea denaturing polyacrylamide gel. After staining the gel with SYBR Gold, RNA was transferred onto a positively charged nylon membrane. The membrane was cross-linked by UV irradiation at 120 mJ/cm^2^ and pre-hybridized with ULTRAhyb-Oligo Ultrasensitive Hybridization Buffer (Thermo Fisher Scientific, Waltham, MA, USA, AM8670). Biotin-labeled LNA-modified DNA probes (synthesized by Jiangsu Saisofei, Wuxi, China) were added at a concentration of 50 pmol/mL and hybridized overnight at 37 °C. Following sequential washes with low-stringency buffer, high-stringency buffer, and 1× SSC, the membrane was processed and developed using the Chemiluminescent Nucleic Acid Detection Module Kit (Beyotime, Shanghai, China, D3308), with 5S rRNA serving as the internal control. Probe sequences were as follows: 5S rRNA, 5′-Biotin-AAAGCCTACAGCACCCGGTATTCCC-3′; BZL-sRNA-20, 5′-Biotin-gAtCgtCGGaCtGtAgaaCtCtGaAcc-3′ (uppercase letters indicate LNA-modified positions).

### 2.12. Statistical Analysis

Data were analyzed with GraphPad Prism 9.5 and are presented as the mean ± SD of at least three independent experiments (N). Statistical significance was set at *p* < 0.05 and is indicated as * *p* < 0.05, ** *p* < 0.01, *** *p* < 0.001, **** *p* < 0.0001; *ns*, not significant.

## 3. Results

### 3.1. In Vitro-Transcribed 5′-PPP miRNAs Activate the RIG-I Pathway

**Figure 1:**
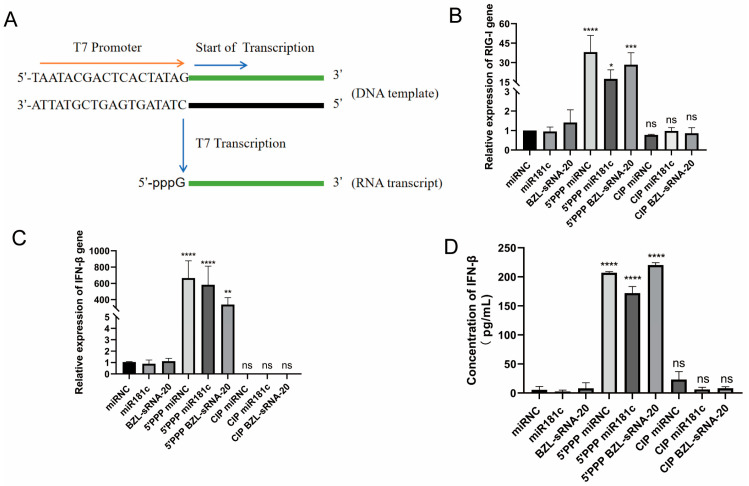
In vitro-transcribed 5′-PPP miRNAs activate the RIG-I pathway. (**A**) Schematic of 5′-PPP miRNA synthesis by in vitro transcription. (**B**–**D**) MARC-145 cells were transfected with unmodified, 5′-PPP, or CIP-treated miRNAs (20 nM final) using TransIT-X2. After 48 h, total RNA was extracted and subjected to RT-qPCR analysis of RIG-I and IFN-β mRNA expression (normalized to β-actin), while IFN-β protein levels in culture supernatants were measured by ELISA. Data are presented as mean ± SD from three independent experiments (N = 3). Statistical significance was determined by one-way ANOVA followed by Tukey’s post hoc test. * *p* < 0.05, ** *p* < 0.01, *** *p* < 0.001, **** *p* < 0.0001; *ns*, not significant.

This study aimed to create bifunctional RNA that couples innate immune activation with viral gene silencing. We selected two mechanistically distinct miRNAs: miR-181c, which blocks PRRSV entry by targeting the host CD163 receptor [23], and BZL-sRNA-20, a plant derived broad spectrum sRNA that targets the host TLR4 pathway and attenuates virus-induced cytokine storms [10]. Both candidates were synthesized by in vitro transcription to carry a 5′-triphosphate (5′-PPP) group essential for RIG-I recognition (Figure 1A). As controls, we generated non-phosphorylated counterparts and CIP-treated RNAs in which the 5′-PPP group was enzymatically removed.

To verify that the 5′-PPP modification is indispensable for RIG-I activation, these RNA variants were transfected into MARC-145 cells. RT-qPCR and ELISA analyses revealed that 5′-PPP-modified miR-181c and BZL-sRNA-20, but not their unmodified or miR-NC counterparts, dramatically up-regulated RIG-I and downstream IFN-β mRNA and protein levels (Figure 1B–D). This potent immune stimulation was completely abolished after CIP-mediated removal of the 5′-PPP group, restoring activity to baseline levels comparable to those of unmodified miRNAs. Collectively, these findings demonstrate that our engineered 5′-PPP-miRNAs are specifically recognized by RIG-I and effectively initiate the interferon-signaling cascade, with activation strictly dependent on the 5′-triphosphate moiety.

### 3.2. 5′-PPP BZL-sRNA-20 Potently Inhibits PRRSV Replication In Vitro

**Figure 2:**
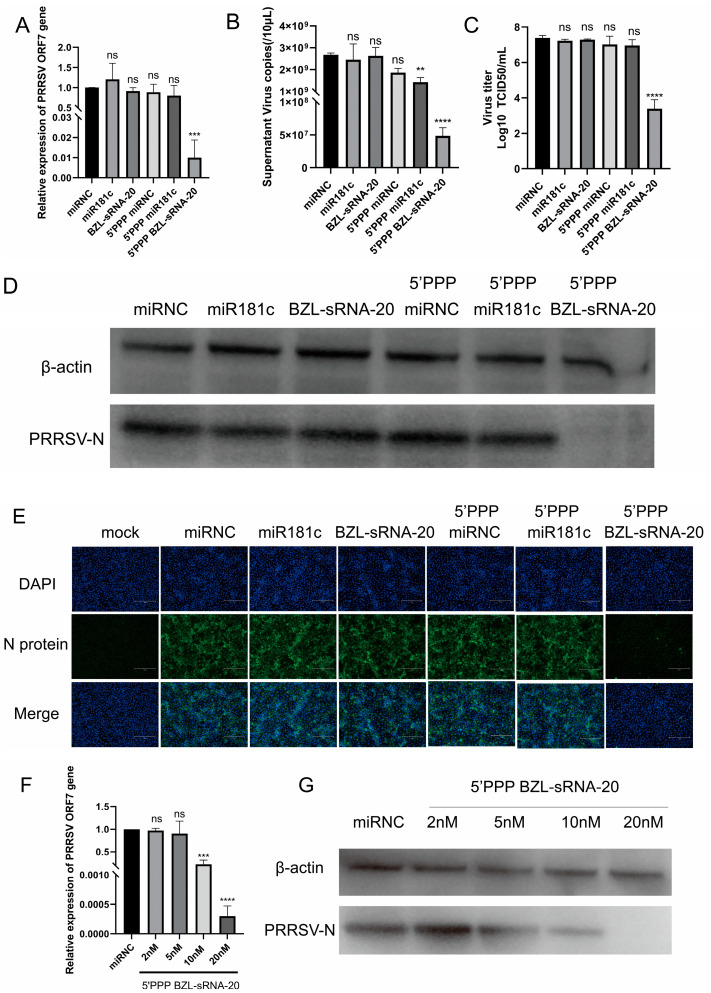
5′-PPP BZL-sRNA-20 potently suppresses PRRSV replication in vitro. (**A**–**C**) MARC-145 cells were transfected with unmodified or 5′-PPP miRNAs (20 nM final) using TransIT-X2, challenged 24 h later with PRRSV NADC30-like (MOI = 0.1), and harvested at 24 h post-infection. Intracellular ORF7 mRNA was quantified by RT-qPCR; genome copies in supernatants were calculated from a standard curve generated by serial dilution of an *ORF7*-containing plasmid, and infectious titers were determined by TCID_50_ using the Reed-Muench method. (**D**,**E**) Parallel cultures processed identically were analyzed for viral N protein by Western blot and immunofluorescence. Scale bar = 300 μm. (**F**,**G**) Dose–response: cells transfected with graded concentrations of 5′-PPP BZL-sRNA-20 (2–20 nM) or miR-NC were infected 24 h later; ORF7 mRNA and protein were assessed at 24 h by RT-qPCR and Western blot, respectively. ** *p* < 0.01, *** *p* < 0.001, **** *p* < 0.0001; *ns*, not significant.

Having established that the 5′-PPP modification efficiently triggers RIG-I signaling, we next evaluated the antiviral efficacy of these engineered miRNAs in a PRRSV infection model. MARC-145 cells were first transfected with unmodified or 5′-PPP-modified miRNAs, followed by infection with PRRSV. Among all treatments, only 5′-PPP BZL-sRNA-20 potently inhibited viral replication. Compared with the negative-control group, this molecule reduced intracellular viral *ORF7* mRNA by approximately 100-fold (Figure 2A) and decreased viral genome copies in the supernatant by about 55-fold (Figure 2B). Consistent with these results, TCID_50_ assays revealed a substantial reduction in infectious viral titers following treatment with 5′-PPP BZL-sRNA-20 (Figure 2C). In contrast, 5′-PPP miR-181c only slightly lowered supernatant viral load, whereas unmodified BZL-sRNA-20 and miR-181c showed no significant effect, indicating that RIG-I activation alone is insufficient to suppress PRRSV and that efficient genome targeting is essential for achieving robust antiviral effects.

Western blot and immunofluorescence analyses corroborated these findings: viral N protein was almost undetectable in the 5′-PPP BZL-sRNA-20 group but clearly present in all other groups (Figure 2D,E). Moreover, the inhibitory effect was dose-dependent: increasing transfection concentrations progressively reduced both intracellular *ORF7* mRNA and N protein levels (Figure 2F,G).

Collectively, these data demonstrate that 5′-PPP BZL-sRNA-20 effectively suppresses PRRSV replication in vitro. The superior antiviral performance suggests a synergistic interplay between RIG-I-mediated innate activation and the intrinsic targeting capacity of BZL-sRNA-20, prompting us to next dissect its precise targeting mechanism.

### 3.3. 5′-PPP BZL-sRNA-20 Exerts Antiviral Activity by Directly Targeting the Viral Genome

Our preceding analyses revealed that 5′-PPP BZL-sRNA-20 exerts markedly stronger antiviral activity than 5′-PPP miR-181c, strongly implying that the intrinsic targeting capacity of BZL-sRNA-20 is pivotal. To determine whether this molecule acts directly on the viral genome, we first performed a genome-wide target prediction using RNAhybrid, which identified 11 potential binding sites for BZL-sRNA-20 within the PRRSV genome (Figure S2).

**Figure 3:**
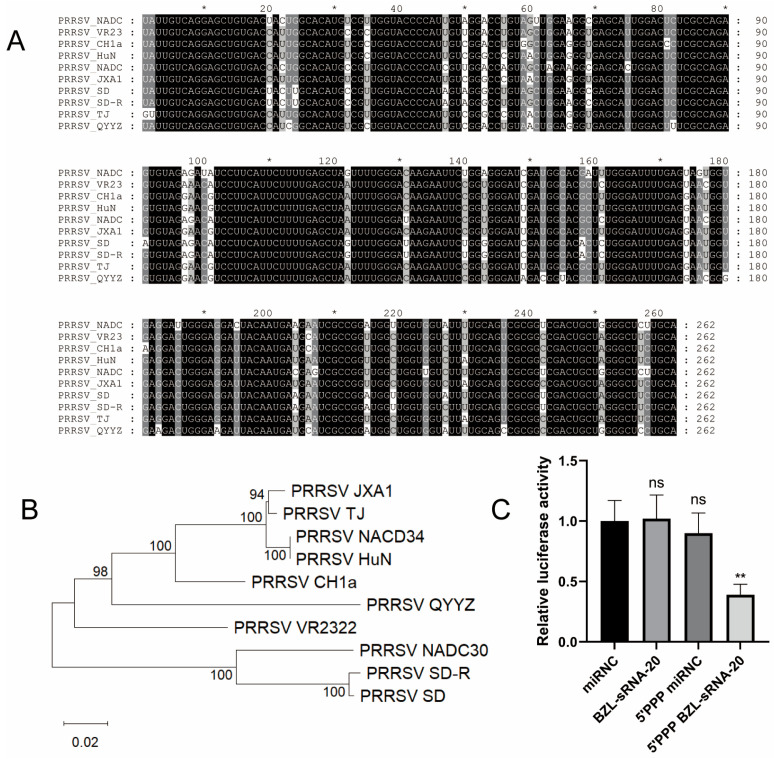
5′-PPP BZL-sRNA-20 directly targets the PRRSV genome. (**A**) PRRSV genomic sequences encompassing BZL-sRNA-20 target sites were retrieved from NCBI; shading intensity reflects nucleotide divergence. Asterisks (*) are automatic alignment markers from MEGA; shading indicates nucleotide divergence. (**B**) A maximum-likelihood phylogenetic tree was constructed with MEGA 12.0 to illustrate the genetic relationships among the selected strains. Bootstrap values (1000 replicates) are indicated at the nodes. (**C**) MARC-145 cells were co-transfected with pmirGLO-PRRSV and the indicated miRNAs (miR-NC, unmodified BZL-sRNA-20, or 5′-PPP BZL-sRNA-20). Dual-luciferase activities were measured 48 h post-transfection. ** *p* < 0.01; *ns*, not significant.

Given the high variability in RNA viruses, the conservation of target sequences is essential for broad-spectrum, escape-resistant therapeutics. We therefore retrieved complete genome sequences of representative PRRSV strains covering different lineages from NCBI, conducted multiple sequence alignments to assess the conservation of all 11 predicted sites, and constructed a phylogenetic tree to illustrate the genetic diversity of the selected isolates (Figure 3B). Nine of the eleven predicted binding sites were found to be highly conserved across the analyzed strains (Figure 3A); two poorly conserved sites were excluded to minimize the risk of viral escape through target-site mutation.

To experimentally validate these predicted interactions, the nine conserved sites were concatenated in genomic order and cloned into the pmirGLO dual-luciferase reporter vector to generate pmirGLO-PRRSV. Co-transfection of this reporter with individual miRNAs into MARC-145 cells revealed that only 5′-PPP BZL-sRNA-20 significantly suppressed luciferase activity, whereas unmodified BZL-sRNA-20 and the negative control 5′-PPP miR-NC had no effect (Figure 3C). Notably, the strict dependence of silencing activity on the 5′-PPP modification was unusual, as standard miRNA-mediated gene silencing typically requires only a 5′-monophosphate. To investigate whether this “atypical” phenomenon stemmed from differences in RNA stability, we performed Northern blot analysis of transfected cells. At 24 and 48 h post-transfection, the signal intensity of 5′-PPP BZL-sRNA-20 remained robust, whereas unmodified BZL-sRNA-20 was barely detectable at 24 h and completely degraded by 48 h (Figure S3), indicating that the 5′-PPP moiety confers resistance to 5′→3′ exonuclease-mediated degradation. Thus, the observed differences in luciferase activity primarily result from greater accumulation of functional sRNA molecules.

In summary, this mechanistic study clarifies that the potent antiviral activity of 5′-PPP BZL-sRNA-20 rests on two integrated functions: (i) its 5′-PPP terminus acts as an “immune-activation switch” that efficiently triggers the RIG-I pathway; (ii) its RNA sequence serves as a “precision guidance system” that directly cleaves the viral genome. The synergistic integration of these dual activities—enabled by the stabilizing effect of the 5′-PPP modification—underlies the exceptional efficacy of 5′-PPP BZL-sRNA-20 against PRRSV.

### 3.4. miR-181c and 5′-PPP miRNAs Suppress PRRSV Replication in PAM Cells

Although experiments in MARC-145 cells established that 5′-PPP BZL-sRNA-20 exerts antiviral effects through synergistic “immune activation” and “targeted silencing”, PRRSV primarily infects porcine alveolar macrophages (PAMs) in vivo. These cells possess an intact innate immune repertoire and represent a physiologically relevant model for evaluating therapeutic potential. We therefore extended our analysis to this primary cell system.

**Figure 4:**
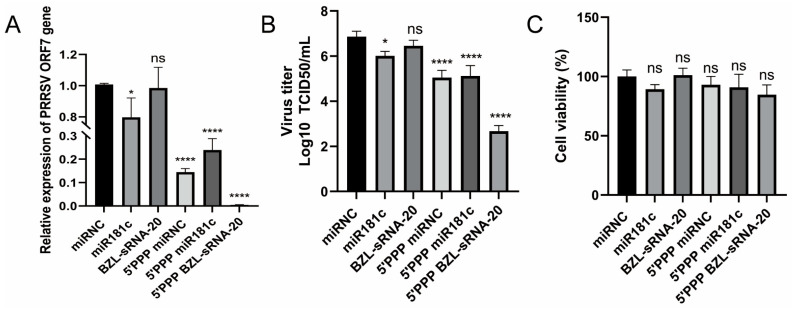
miR-181c and 5′-PPP miRNAs inhibit PRRSV replication in PAM cells. (**A**,**B**) PAMs were transfected with the indicated miRNAs (20 nM) using TransIT-X2, challenged 24 h later with PRRSV NADC30-like (MOI = 0.1), and harvested at 24 h post-infection. Intracellular *ORF7* mRNA was quantified by RT-qPCR, and infectious titres in supernatants were determined by TCID_50_ assay. (**C**) Cell viability of PAMs treated under the same conditions as in (**A**,**B**) was measured at 24 h post-infection using the CCK-8 assay (absorbance at 450 nm). Data are presented as mean ± SD from N = 3 independent experiments. Statistical significance was determined by one-way ANOVA followed by Tukey’s post hoc test. Note: In PAMs, effects are primarily driven by immune activation (see Discussion). * *p* < 0.05, **** *p* < 0.0001; *ns*, not significant.

In contrast to MARC-145 cells—where only 5′-PPP BZL-sRNA-20 was effective—all 5′-PPP-modified miRNAs exhibited significant antiviral activity in PAMs (Figure 4A,B). Consistent with earlier findings, 5′-PPP BZL-sRNA-20 remained the most potent inhibitor. This discrepancy suggests that in PAMs, the 5′-PPP-mediated “immune-activation” signal alone establishes a robust basal antiviral state, enabling even the less efficiently targeted 5′-PPP miR-181c to suppress viral replication.

To rule out the possibility that the observed antiviral effects resulted from cytotoxicity rather than genuine therapeutic activity, we further assessed cell viability under identical experimental conditions. Cell Counting Kit-8 (CCK-8) assays revealed that all treatment groups maintained high viability (>85%) comparable to that of the control group, with no statistically significant differences observed among of the miRNA constructs (Figure 4C). These data demonstrate that the robust antiviral state induced by 5′-PPP-modified RNAs does not compromise cell survival, confirming that the observed suppression of viral replication reflects authentic therapeutic efficacy rather than non-specific cytotoxicity or apoptosis.

Interestingly, unmodified miR-181c also produced a modest reduction in viral load in PAMs, consistent with its known mechanism of blocking viral entry by targeting the CD163 receptor [19]. Thus, in this complex cellular environment, our bifunctional strategy displays multi-layered synergy: immune activation (5′-PPP module) combined with either blockade of viral entry (miR-181c module) or cleavage of the viral genome (BZL-sRNA-20 module) cooperatively interrupts multiple steps of the viral life cycle.

Collectively, the results obtained in the natural target cells of PRRSV provide compelling evidence that our engineered bifunctional miRNA strategy is even more advantageous in an immunocompetent context. Activation of innate immunity not only contributes directly to antiviral efficacy but also appears to potentiate the intrinsic inhibitory activities of different miRNAs, offering a convincing experimental basis for achieving more efficient and durable control of PRRSV.

## 4. Discussion

The immunosuppressive nature of and high genetic variability in porcine reproductive and respiratory syndrome virus (PRRSV) underlie the limited efficacy of conventional vaccines and single-target therapeutics [24,25]. Although miRNA-based gene silencing offers a promising avenue for antiviral therapy [23,26], its efficacy as monotherapy is often compromised by viral escape [11,12]. In contrast, activation of innate immune pathways through 5′-triphosphate (5′-PPP) RNA can elicit broad-spectrum antiviral responses, but such activation lacks pathogen specificity [16,17,18]. Here we propose and validate, for the first time, an “immune-activation plus gene-silencing” antiviral paradigm. By rationally engineering a bifunctional miRNA that integrates the potent 5′-PPP “immune switch” with a sequence-specific silencing module, we simultaneously overcome PRRSV-driven immunosuppression and the escape hurdle.

Our study establishes a paradigm of “immune activation coupled with precision targeting” that addresses these limitations through the rational engineering of bifunctional miRNAs. However, the relative contribution of these mechanisms is context-dependent and varies across cell types. In MARC-145 cells, robust antiviral activity was observed only when immune activation and gene silencing were combined in the same construct, highlighting the advantage of this bifunctional design in cells with limited intrinsic immunity. Dual-luciferase assays revealed that the silencing capacity of BZL-sRNA-20 depends on 5′-triphosphate modification. In addition, Northern blot analysis further demonstrated that 5′-PPP modification significantly enhances RNA stability (Figure S3). These observations suggest that silencing activity depends on 5′-PPP modification, which enhances RNA stability and prolongs intracellular persistence, thereby facilitating efficient engagement of the RNAi machinery. Accordingly, the reduced silencing observed in the absence of the 5′-PPP reflects insufficient RNA stability and persistence rather than an intrinsic defect in the silencing capacity of BZL-sRNA-20.

Most importantly, validation in PAMs—the natural host cells for PRRSV—revealed a distinct mechanism. In these cells, 5′-PPP modification achieved antiviral effects primarily through potent interferon responses, with the targeting sequence providing limited or context-dependent benefits. This indicates that in physiologically relevant cells, the “immune activation” component dominates, while the contribution of sequence-specific silencing, though present, remains subordinate. Thus, rather than indicating universal synergy, our data support a model in which the 5′-PPP acts as the primary driver of antiviral efficacy through RIG-I activation and RNA stabilization, while sequence-specific targeting confers additional specificity and limits viral escape in a context-dependent manner.

Compared with conventional single-mechanism approaches, this bifunctional strategy offers several potential advantages: (i) the activation of the RIG-I pathway directly counteracts PRRSV-induced immunosuppression, thereby restoring the host cell’s intrinsic antiviral defenses; (ii) by simultaneously targeting the viral genome, it not only eliminates virus but also raises the genetic barrier to escape—an effect most evident in cell lines where both mechanisms operate efficiently, such as MARC-145. Analysis of conserved targets (Figure 3A,B) underpins broad-spectrum applicability, though the relative importance of targeting versus immune activation may vary by infection context.

Despite these promising findings, the therapeutic application of 5′PPP-BZL-sRNA-20 in vivo will require careful consideration of RNA stability and delivery strategies. Naked RNA molecules are rapidly degraded by circulating ribonucleases in serum, and although the 5′-triphosphate structure confers exonuclease resistance, additional chemical modifications are necessary to further enhance stability. Future optimization may incorporate modifications such as 2′-O-methylation, phosphorothioate backbone substitutions, at critical positions to enhance nuclease resistance and prolong RNA half-life. Given that PRRSV primarily infects porcine alveolar macrophages (PAMs), lipid nanoparticle (LNP) encapsulation systems can be optimized by adjusting ratios of ionizable cationic lipids and helper lipids to achieve specific enrichment in pulmonary macrophages. Moreover, conjugation with antibodies against CD163 or CD169, or modification with PAM-specific aptamers, would enable precise delivery to viral infection sites.

In addition to its therapeutic potential, the rapid immune-activating property of 5′PPP-miRNA makes it highly attractive for prophylactic applications. Unlike conventional vaccines, which typically require weeks to establish adaptive immunity, 5′PPP-miRNA can trigger an immediate antiviral state within hours via the RIG-I pathway. To translate this into a practical strategy, future studies will evaluate its efficacy in pigs against PRRSV challenge. This could be achieved by integrating expression cassettes for 5′PPP-miRNAs into oral delivery platforms, such as engineered yeast or probiotics, and assessing the resulting antiviral protection. Furthermore, 5′PPP-miRNA could be administered as an adjunct to commercial vaccines to provide immediate coverage during the window period before vaccine-specific immunity develops, potentially reducing peak viremia and pulmonary pathology. The safety and prophylactic efficacy of this approach would be assessed by monitoring clinical parameters, including body temperature, viral load, lung pathology, and cytokine profiles. This dual-mode strategy, combining immediate innate immune control with eventual adaptive protection, represents a novel and comprehensive approach for PRRSV prevention and control.

Finally, the design principle described here may represent a broadly adaptable platform for RNA-based antiviral therapeutics. By simply replacing the miRNA guide sequence, the “immune-activation module” can be paired with silencing modules directed against other viral pathogens or host factors involved in viral replication. The stability-enhancing effect of 5′-PPP observed in our Northern blot data also suggests that this modification confers universal benefits for RNA-based therapeutics beyond RIG-I activation, further supporting its utility in antiviral drug design.

Collectively, our findings establish 5′-PPP miRNA as a promising bifunctional antiviral strategy that reconciles the conflicting requirements of broad immune activation and precise pathogen targeting. By harnessing the dual mechanisms of RIG-I pathway reactivation and sequence-specific silencing, this approach offers a robust solution to the challenges posed by PRRSV and potentially other immunosuppressive viruses.

## 5. Patents

A related patent on triple phosphorylated miRNA and its application in antiviral therapy is currently under application (Application No. 202512058706.4).

## Figures and Tables

**Table 1:** qPCR Primer Sequences.

Primer Names	Sequences (5′→3′)
β-actin-F	GGCATCCATGAAACTACCTTC
β-actin-R	AGGGCAGTAATCTCCTTCTG
RIG-I-F	CTGACTGCCTCGGTTGGTGTTG
RIG-I-R	CTCCAGTTCCTCCAGGTTGTCTTTG
IFNβ-F	TGCTCTCCTGTTGTGCTTCTCC
IFNβ-R	CATCTCATAGATGGTCAATGCGG
ORF7-F	CAGCCAGTCAATCAGCTGTGCCA
ORF7-R	AGAGGAAAATGGGGCTTCTC

## Data Availability

The original contributions presented in this study are included in the article/Supplementary Material. Further inquiries can be directed to the corresponding authors.

## Supplementary Materials

The following supporting information can be downloaded at https://www.mdpi.com/article/10.3390/v18030390/s1. Figure S1: Phylogenetic Analysis of the GP5 gene of PRRSV strain B39; Figure S2: Predi ct ion of BZL-sRNA-20 targeting NADC30-like PRRSV genes; Figure S3: 5′-Triphosphate modification enhances the stability of BZL-sRNA-20 in cells; Table S1: Unmodified miRNA mimics sequence.
