# Supplementary material for: Synergistic Inhibition of Porcine Reproductive and Respiratory Syndrome Virus by a Bifunctional 5′-PPP miRNA Combining RIG-I Activation with Sequence-Specific Viral Targeting
Source: Viruses. 2026 Mar 20;18(3):390. doi: 10.3390/v18030390 (PMC13030578; doi:10.3390/v18030390)
Supplement: Supplementary file 1 [file viruses-18-00390-s001.zip › viruses-4145925-supplementary.pdf]

viruses

MDPI

Supplementary Materials:

Table S1. Unmodified miRNA mimics sequence

miRNA mimics	sense (5′→3′)	antisense (5′→3′)
miR-NC	UUCUCCGAACGUG-UCACGUTT	ACGUGACACGUUCGGA-GAATT
miR-181c	AACAUUCAAC-CUGUCGGUGAGU	UCAC-CGACAGGUUGAAU-GUUUU
BZL-sRNA-20	GUUCAGAGUUCUACAGU CCGACGAUC	UCGUCGGACUGUAGA-ACUCUGAACUU

Figure S1

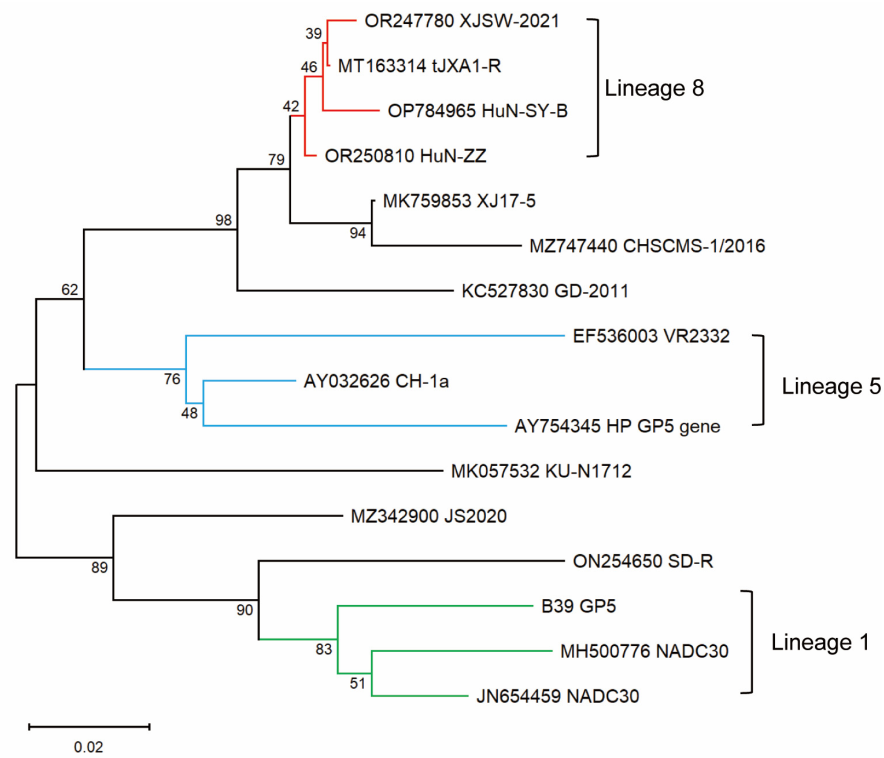

Figure S1 Phylogenetic Analysis of the GP5 gene of PRRSV strain B39.
The phylogenetic tree was constructed by the maximum-likelihood method using MEGA 12.0 based on the GP5 gene nucleotide sequences of strain B39 and reference strains. Bootstrap values (1000 replicates) are indicated at the nodes. Strain B39 clusters with NADC30 reference strains (JN654459 and MH500776) in Lineage 1, confirming its identity as the NADC30-like genotype. Lineages 5 and 8 are shown as controls to illustrate genetic divergence. The scale bar indicates nucleotide substitutions per site.

Figure S2

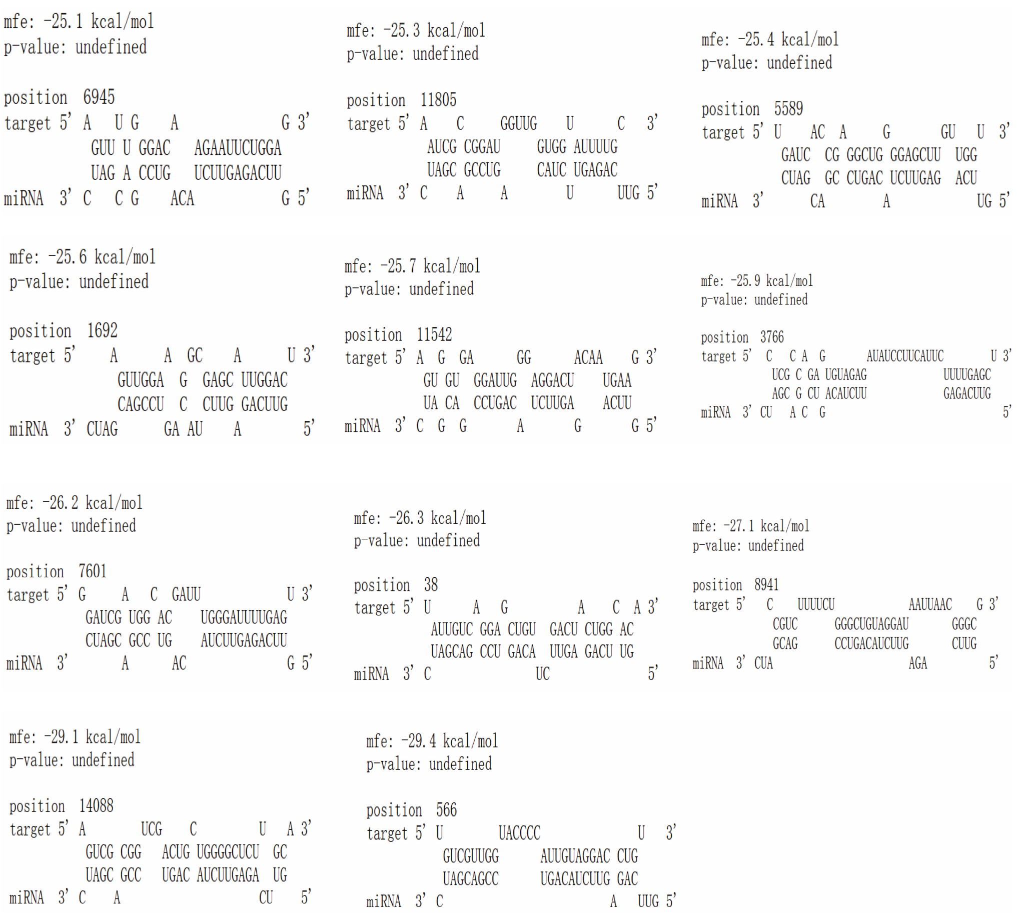

Figure S2 Prediction of BZL-sRNA-20 targeting NADC30-like PRRSV genes.
Alignment results of the prediction analysis for BZL-sRNA-20 targeting the NADC30-like PRRSV genome, performed with RNAhybrid.

Figure S3

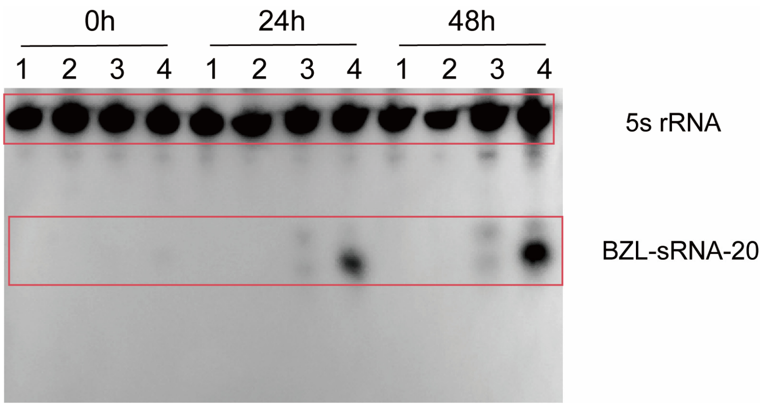

Figure S3 5′-Triphosphate modification enhances the stability of BZL-sRNA-20 in cells.
MARC-145 cells were transfected with the indicated RNAs (20 nM), and total RNA was extracted at 0, 24, and 48 h post-transfection for Northern blot analysis. 5S rRNA (upper panel) served as a loading control. 1, miR-NC; 2, 5′-PPP miR-NC; 3, BZL-sRNA-20; 4, 5′-PPP BZL-sRNA-20.
